# The dynamic inflammatory profile of pregnancy can be monitored using a novel lipid-based mass spectrometry technique†

**DOI:** 10.1039/d2mo00294a

**Published:** 2023-03-08

**Authors:** April Rees, Zoe Edwards-I-Coll, Oliver Richards, Molly E Raikes, Roberto Angelini, Catherine A Thornton

**Affiliations:** a Institute of Life Science, Swansea University Medical School Swansea Wales UK SA2 8PP c.a.thornton@swansea.ac.uk +44 (0)1792 602122

## Abstract

The lipid environment changes throughout pregnancy both physiologically with emergent insulin resistance and pathologically *e.g.*, gestational diabetes mellitus (GDM). Novel mass spectrometry (MS) techniques applied to minimally processed blood might lend themselves to monitoring changing lipid profiles to inform care decisions across pregnancy. In this study we use an intact-sandwich, MALDI-ToF MS method to identify phosphatidylcholine (PC) and lysophosphatidylcholine (LPC) species and calculate their ratio as an indicator of inflammation. Plasma and sera were prepared from venous blood of non-pregnant women (aged 18–40) and pregnant women at 16 weeks, 28 weeks (including GDM-positive women), and 37+ weeks (term) of gestation alongside umbilical cord blood (UCB). Women with a normal menstrual cycle and age-matched men provided finger-prick derived capillary sera at 6 time-points over a month. Serum rather than plasma was preferable for PC/LPC measurement. As pregnancy progresses, an anti-inflammatory phenotype dominates the maternal circulation, evidenced by increasing PC/LPC ratio. In contrast, the PC/LPC ratio of UCB was aligned to that of non-pregnant donors. BMI had no significant effect on the PC/LPC ratio, but GDM-complicated pregnancies had significantly lower PC/LPC at 16 weeks of gestation. To further translate the use of the PC/LPC ratio clinically, the utility of finger-prick blood was evaluated; no significant difference between capillary *versus* venous serum was found and we revealed the PC/LPC ratio oscillates with the menstrual cycle. Overall, we show that the PC/LPC ratio can be measured simply in human serum and has the potential to be used as a time-efficient and less invasive biomarker of (mal)adaptative inflammation.

## Abstract 2

The lipid environment changes throughout pregnancy both physiologically with emergent insulin resistance and pathologically *e.g.*, gestational diabetes mellitus (GDM). Made with BioRender.

## Graphical abstract

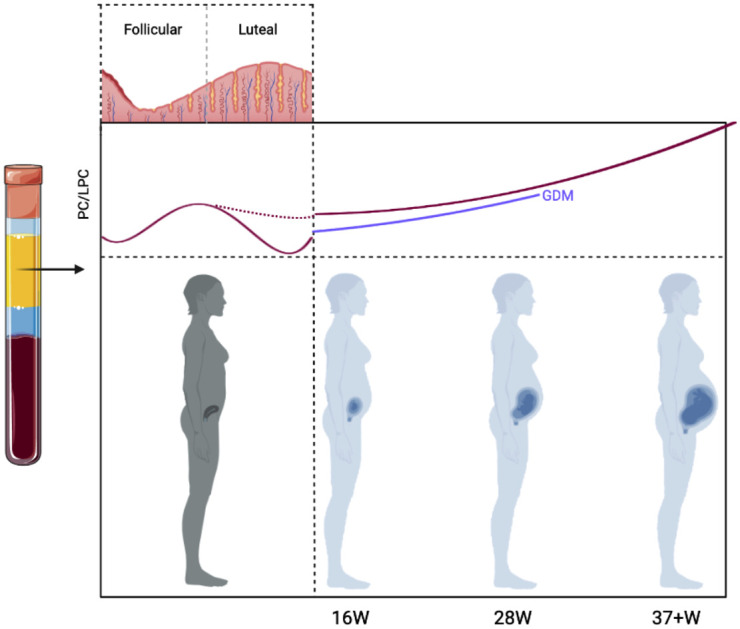

## Introduction

Lipidomics, the large-scale study of lipids, is of immense interest due to the realisation that lipids are involved in more than membrane structure and energy production. Lipids are now also recognised for their role in signal transduction, regulation of membrane proteins, vesicular trafficking, cytoskeletal rearrangements, and secretion. Because of these vital roles, disruption of lipid metabolism is involved in various pathologies, such as neurological disorders,^1^ cancer,^2^ and cardiovascular diseases.^3^

Phosphotidylcholines (PC) are a class of phospholipids that are a major component of cells where they are vital in supporting membrane bilayers and can be found systemically in the blood bound to lipoproteins. PC itself is produced from phosphatidylethanolamine (PE) *via* three sequential methylations by *S*-adenosyl methionine (SAM) catalysed by phosphatidylethanolamine *N*-methyltransferase (PEMT). PC can also be converted from choline obtained by dietary consumption or metabolism of lipids which contain choline. Cleavage of PC by phospholipase A_2_ (PLA_2_) produces lysophosphotidylcholine (LPC) with free-fatty acids, such as polyunsaturated fatty acids (PUFAs) which are the precursors for eicosanoids, released as by-products. This generates a variety of downstream cellular effectors, with the lipid mediators produced being involved in both pro- (*e.g.*, prostaglandins^4–6^) and anti- (*e.g.*, resolvins^7–10^) inflammatory processes. Inflammation describes the response to a pathogen, tissue damage or injury that presents with the hallmarks of swelling, pain, heat, redness, and loss of function and is driven by various cellular (*e.g.*, macrophages, neutrophils and eosinophils) and soluble mediators (*e.g.*, lipid mediators and cytokines) some of which will be pro-inflammatory and drive the occurrence, maintenance and/or exacerbation of inflammation and others that are anti-inflammatory and serve to dampen this response and support resolution and repair. Many chronic diseases are characterised by inflammation driven by many of the same cell types and soluble mediators, but it occurs in a specific tissue and underpins loss of or altered function of that tissue.

Oxidised low-density lipoproteins (oxLDL) are rich in LPC and when the LPC is released by apoptotic cells this stimulates the recruitment of phagocytes^11^ by stimulating the expression of chemoattractant molecules such as monocyte chemoattractant protein 1 (MCP-1);^12^ high levels of LPC are therefore synonymous with inflammation and have been observed in inflammatory diseases such as atherosclerosis.^13^ LPCs have been implicated in the demyelination observed in autoimmune disorder multiple sclerosis (MS),^14^ as they have been shown to stimulate phagocytosis of the myelin sheath by macrophages.^15^ The secretion of prostaglandins (which are also a by-product from the production of LPC from PC by phospholipase A2) from peripheral monocytes predict relapses, and clinical activity is accompanied by increased activation markers and release of IL-1 and TNFα.^16^ In addition, LPC can interact with LOX1, lipid flippase and G protein-coupled receptor signalling to exert harmful effects on cells.^17^ LPC can also be further converted into lipoprotein(a) (LPA) of which levels are associated with atherosclerosis,^18^ stroke^19^ and coronary heart disease.^20^ The metabolism of PC to LPC, measurable as a PC/LPC ratio, then is vital as a potential marker of disease-associated inflammation. Notably, this ratio is not simply a measure of PLA_2_ activity. While LPC is derived predominantly from PC by PLA_2_ in the circulation, the inability of LPC to be retaken up into tissue by lysophospatidylcholine acyl transferase 1 (LPCAT) can also contribute to raised circulating LPC levels. LPC can also be degraded in the circulation by lysophospholipases^17^ and the secretion of lectin-cholesterol acyltransferase (LCAT) by the liver catalyses PC to cholesterol in plasma and results in LPC formation on HDL and LDL.^21^

Pregnancy is associated with various metabolic adaptations in order to facilitate fetal growth and development.^22^ To supply the fetus with glucose, maternal glucose production and intolerance, and insulin resistance occurs,^23^ with a transition from lipid storage to lipolysis necessary to fuel the mother's own energy demands.^24^ In late pregnancy, this is evidenced by increased levels of postprandial free fatty acids (FFA) accompanied by decreased maternal adipose tissue deposits^25^ that were expanded *via* lipogenesis in the first two trimesters for this purpose.^26,27^

Lipoproteins, a combination of various types of proteins and fats, are necessary for the transport of triglycerides and cholesterol through the blood. During pregnancy the synthesis of triglycerides is increased by (40% at 18 weeks of gestation and by 250% at term^26,28^), (cholesterol levels are increased by 50% at term^26,28^). This is accompanied by a net increase in lipoproteins: high-density lipoprotein (HDL) levels increase progressively until 24 weeks of pregnancy before decreasing until 32 weeks where they plateau,^26^ low-density lipoprotein (LDL). Levels undergo a small decrease during the initial stages of pregnancy before rising steadily,^26^ and very-low-density lipoprotein (VLDL) levels triple during the second and third trimester. PCs are a major component of lipoproteins yet despite the knowledge about changing lipoprotein profiles with pregnancy, there is sparingly little data regarding phospholipid levels and their trends at this time.

As pregnancy is accompanied naturally by altered inflammatory status,^29–31^ the monitoring of the PC/LPC ratio might serve as both a tool to monitor this normal fluctuation of inflammation and reveal dysregulated inflammation associated with multiple adverse obstetric outcomes such as gestational diabetes mellitus (GDM) and preeclampsia. Studies have reported elevated serum PC in the risk of developing GDM independently of maternal BMI,^32–34^ increased plasma LPC in preterm delivery,^35^ and reduced serum PC in preeclampsia.^36^ However, traditional methods for the determination of lipid content, including for calculation of the PC/LPC ratio, are convoluted and time-consuming, requiring lipids be extracted before analysis with mass spectrometry. Using horse serum, Angelini *et al.* have developed and validated using quantitative nuclear magnetic resonance (NMR) a simple method to analyse ‘intact’ samples (*i.e.*, no processing of the sample required) with matrix assisted laser desorption/ionisation (MALDI) time of flight (ToF) mass spectrometry (MS) to quickly and accurately determine the PC/LPC ratio.^37^

The use of novel and more time-efficient techniques to determine changes in PC/LPC ratios in minimally processed blood, would allow determination of alterations in gross inflammatory status with potential clinical utility. Firstly, suitability of this intact serum analysis approach in humans needs to be confirmed and a range for healthy pregnancy established. Here, we show a gestational trend in the PC/LPC ratio, with the possibility of usage as a biomarker for GDM during early gestation. To facilitate diagnostic translation, we then go onto confirm that this ratio can be calculated using capillary blood serum from finger-pricks and used to track normal fluctuation in the menstrual cycle.

## Materials & methods

### Human sample collection

Venous blood was collected into one heparinised and one serum-activating Vacuette™ (Greiner Bio-one, Frickenhausen, Germany) from healthy non-pregnant women (aged 18–40 years), age matched pregnant women at full term (37+ weeks) and venous umbilical cord and processed within 90 minutes of collection. Capillary blood was taken at 6 time points over the course of a month (equivalent to menstrual cycle days 1–3, 6–8, 11–13, 16–8, 21–23, 26-end) from normally cycling females *via* finger-prick into a serum-activating MiniCollect® tube (Greiner Bio-one, Frickenhausen, Germany). Females self-reported a normal menstrual cycle and were not on any hormonal contraception. Males were age-matched to the females and paired to the donation time of the females. All samples were collected with informed written consent, and ethical approval obtained from a Health Research Authority Committee (13/WA/0190 – healthy volunteers; 11/WA/0040 – full term pregnant women and cord blood; 19/LO/0722 – pregnant women undergoing glucose tolerance testing) or the Swansea University Medical School (SUMS) Research Ethics Sub-Committee (RESC) (SUMS-RESC 2022-0023 – finger-prick blood).

BMI (kg m^−2^) is calculated by mass (kg)/height (m)^2^. For participants who were pregnant, pre-pregnancy BMI was noted, and for those who were not pregnant, their BMI on date of participation was calculated.

Heparinised blood was centrifuged at 1800 × *g* for 10 min, with the plasma removed and filtered to remove any residual leukocytes (0.22 μm Millipore), before being archived at −80 °C. Blood collected in a serum-activating Vacuette™ was allowed to clot for 90 min before centrifugation (1800 × *g*, 10 min). Serum was collected and stored at −80 °C.

### Measurement of PC/LPC in intact plasma and serum with MALDI-ToF

Serum/plasma samples were diluted 1 : 2 (v : v) with HPLC-grade water (*e.g.* 10 μl serum/plasma with 20 μl water). A 10 mg ml^−1^ matrix solution of 9-aminoacrinide (9-AA) was produced 1 : 1 (w : v) with 2-propanol/acetonitrile (60/40, v/v). 1 μl of the diluted serum/plasma was spotted onto the MALDI target; once dried, 0.5 μl of the 9-AA matrix solution was spotted on top in a “sandwich method”. Once the solvents had evaporated, samples were analysed directly, and spectra acquired by the MALDI-ToF MS. Mass spectra were acquired on a ultrafleXtreme MALDI TOF mass spectrometer (Bruker Daltonics, Bremen, Germany) equipped with a Smartbeam Nd:YAG laser emitting at 355 nm (2 kHz) and operated in the reflectron mode using delayed pulsed extraction and in the positive polarity. Gated matrix suppression was applied to prevent detector saturation (up to *m*/*z* 400) and each mass spectrum was acquired in the *m*/*z* range of 400–1000. Ten thousand single laser shots were summed to obtain each mass spectrum. The laser fluence was kept about 10% above threshold resulting in an optimum signal-to-noise ratio. A mass resolution of >20 000 (fwhm) was typically achieved for peaks of interest.

Data was imported into R (Version 2022.07.1, RStudio) for pre-processing using the MALDIquant package (Version 1.21, ref. 38), which involved smoothing, background subtraction, peak alignment, intensity calibration, peak binning and filtering. Peaks were identified as specific lipids using their *m*/*z* value with the assistance of LIPID MAPS® (https://www.lipidmaps.org/). Peak intensity was used for analysis and calculated within RStudio©, and the subsequent ratio of PC and LPC, which was calculated as 
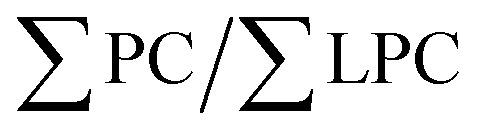
. The peaks and their identifiers for PC and LPC from sera are shown in Table 1.

**Table 1:** The peak *m*/*z* and their assigned molecules as observed in the MALDI-TOF positive ion spectra for human sera

Assignment (positive ion spectra)	Peak *m*/*z*
LPC 16:0 (+H^+^)	496.3398
LPC 16:0 (+Na^+^)	518.3241
LPC 18:0 (+H^+^)	524.2854
LPC 18:0 (+Na^+^)	546.3554
PC 16:0/18:2 (+H^+^)	758.5694
PC 16:0/18:1 (+H^+^)	760.5851
PC 16:0/18:2 (+Na^+^)	780.5538
PC 16:0/18:1 (+Na^+^) and PC 16:0/20:4 (+H^+^)	782.5694
PC 18:0/18:3 (+H^+^)	784.5851
PC 18:0/18:2 (+H^+^)	786.6454
PC 16:0/20:4 (+Na^+^)	804.5749
PC 18:0/18:3 (+Na^+^)	806.5694
PC 18:0/18:2 (+Na^+^)	808.5851

### Statistics

The PC/LPC ratio determined using RStudio©, was predominantly exported onto GraphPad Prism© V9 (Dotmatics) for statistical analysis and data visualisation. Data sets were first tested for normality using the Kolmogorov–Smirnov (K–S) one sample test, where a significant value <0.05 indicated signification deviation from normality. Where the samples were paired, a Wilcoxon matched pairs signed rank test was used. Where only two data sets are analysed and were not paired samples (*e.g.*, healthy *versus* GDM), a Mann–Whitney test was used if the data was non-parametric. In instances where more than two data sets were analysed (*e.g.*, non-pregnant *versus* pregnant *versus* cord), a one-way analysis of variance (ANOVA) with a Tukey *post hoc* test was performed. Depending on whether the data reported as requiring parametric or non-parametric analysis to observe the degree in which two variables correlate (*e.g.*, BMI *versus* PC/LPC) a Pearson or Spearman correlation test was used respectively. The r values are reported to indicate direction (negative values a downward trend; positive values upward trend) and weight of correlation.

To test for covariance, statistical analysis was performed on R studio©. An analysis of covariance (ANCOVA) was used to determine the effect of a continuous variable (BMI) and the effect of a grouping variable (diabetic state and/or stage of gestation). A one-way ANCOVA was used when considering only one grouping variable, and a two-way ANCOVA was used when two independent grouping variables needed to be accounted for. Principal component analysis (PCA) was also performed on RStudio©.

In all statistical analysis, a *p* value < 0.05 was determined to be significant.

The PC/LPC ratio for menstrual cycle analysis was normalised to the maximal value per donor. A polynomial regression analysis was computed using RStudio©. 5 degrees and a *k*-fold cross-validation of 10 was used to test the mean squared error (MSE) of each data set. The lower the MSE, the closer the data points are to a regression line.

## Results

### Serum is chosen over plasma for the determination of PC/LPC

**Fig. 1:**
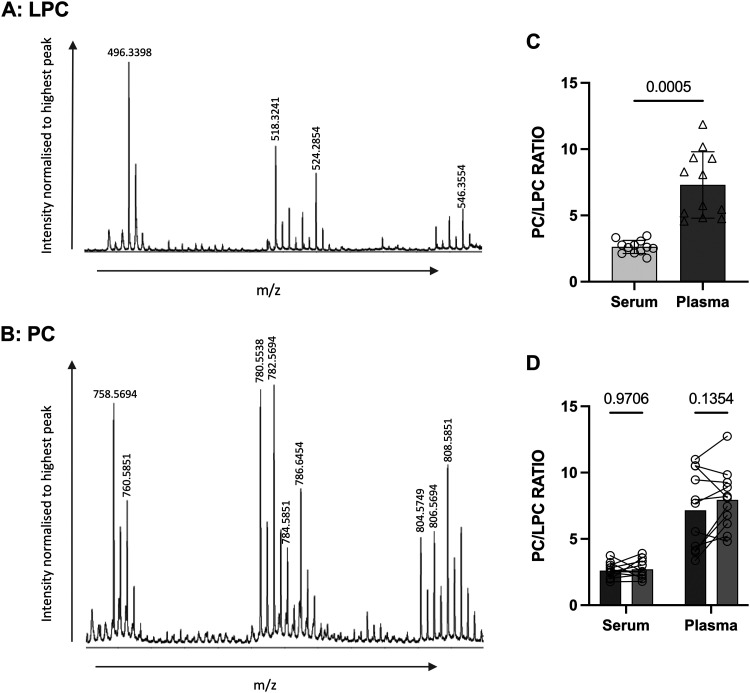
The comparison of plasma *vs.* serum for the determination of the PC/LPC ratio. Matched serum and plasma samples from non-pregnant women (*n* = 12) were analysed using MALDI-ToF for determination of their PC/LPC ratio. Statistical difference was determined using a Wilcoxon test, where *p* < 0.05 was significant. (A) Shows a representation of the human LPC region, whereas (B) illustrates the peaks detectable in the human PC region. (C) The samples for serum (SD = 0.4963) were compared with plasma (SD = 2.511, *p* = 0.0005). (D) The duplicates (which were run at the same time) for serum and plasma were compared for technical reproducibility. Statistical analysis was performed with a 2-way ANOVA using a Šídák's multiple comparison *post hoc* test.

The method for determining the PC/LPC ratio using MALDI-ToF was developed using horse serum^37^ so initially validation of the approach was made using human samples and a comparison of serum *versus* plasma made. Firstly, the LPC and PC species present in human serum and plasma were identified from the horse serum peak list^37^ (Fig. 1(A) and (B) and Table 1). While plasma is typically considered by us and others to be the better choice for studying circulating factors due to the absence of coagulation allowing a closer resemblance to the individual's systemic blood, for the analysis of PC/LPC serum is deemed superior. LPC levels are reported to be increased in serum samples^39^ and plasma must be treated with peptide hydrolytic enzymes such as pepsin to allow for release of LPC from blood lipoproteins for better quantification of LPC.^40^ The use of serum removes the need for treatment with pepsin as the clotting process naturally releases LPC.^39^ As such, the first step was to determine the utility of human serum by comparing it to human heparin-plasma. Samples used for this analysis were matched, *i.e.*, the same blood sample from each non-pregnant donor was used for preparation of serum and plasma in parallel. The PC/LPC ratio was significantly higher in plasma than serum (*p* = 0.0005), with plasma showing greater variability (Fig. 1(C)). In addition, by looking closely at the technical replicates, serum performs significantly better (Fig. 1(D)). Therefore, we opted to progress this work using serum.

### The PC/LPC ratio is altered significantly by gestational age but not by obesity

Archived serum samples from non-pregnant women (aged 18–40 years), age-matched pregnant women (at 16-, 28- or 37+ weeks of gestation) and umbilical cord (from full term deliveries) were then analysed using MALDI-ToF to determine the PC/LPC ratio; 11–15 samples were used for each group.

**Fig. 2:**
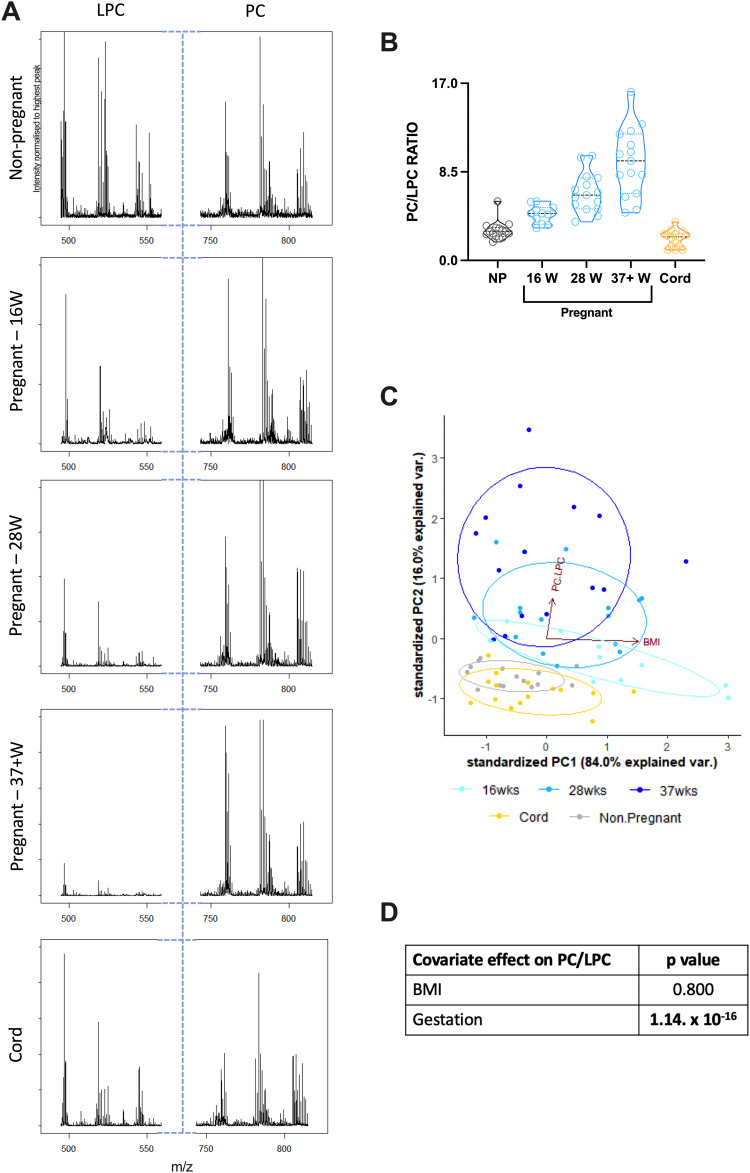
The determination of the PC/LPC ratio throughout pregnancy and in cord blood. Serum samples from non-pregnant (NP; *n* = 15), pregnant (P) at 16 weeks (16W; *n* = 11), 28 weeks (28W; *n* = 15) and term (37 + W; *n* = 15), and umbilical cord (C; *n* = 15), were analysed using MALDI-ToF to determine their PC/LPC ratio. Statistics were performed using an ordinary one-way ANOVA and a Tukey *post hoc* test, where *p* < 0.05 was significant. (A) Example LPC and PC spectra for each group. (B) All groups were compared with each other: NP *vs.* P-16W (*p* = 0.2320), NP *vs.* P-28W (*p* < 0.0001), NP *vs.* P-37 + W (*p* < 0.0001), NP *vs.* C (*p* = 0.7643), P-16W *vs.* P-28W (*p* = 0.0460), P-16W *vs.* P-37 + W (*p* < 0.0001), P-16W *vs.* C (*p* = 0.0182), P-28W *vs.* P-37 + W (*p* = 0.0004), P-28W *vs.* C (*p* < 0.0001), and P-37 + W *vs.* C (*p* < 0.0001). (C) It was then considered if BMI influenced individual samples. For the cord samples, the mother's BMI was taken into consideration. All groups passed the Kolmogorov–Smirnov normality test, and so statistics were determined using the Pearson *r* test, where *p* < 0.05 indicated a significant correlation between BMI and PC/LPC ratio. Groups measured were: NP (*r* = 0.2426, *p* = 0.3836), P-16W (*r* = −0.5890, *p* = 0.0566), P-28W (*r* = 0.08717, *p* = 0.7574), P-37 + W (*r* = 0.1364, *p* = 0.6278) and C (*r* = 1464, *p* = 0.6025). A PCA was performed to determine the explanation of the variance was due to gestational age or BMI. (D) The covariate effect was calculated, and only the gestational age had a significant effect on the PC/LPC ratio.

The PC/LPC ratio was found to increase significantly as gestational age increased (Fig. 2(A) and (B)). This indicates a relative decline in LPC suggesting that the maternal circulation is becoming more anti-inflammatory as pregnancy progresses. Serum prepared from term umbilical cord blood has a PC/LPC ratio like that of non-pregnant adults rather than of pregnant women (Fig. 2(B)).

Given obesity is characterised by low grade systemic inflammation,^41^ the effects of BMI on PC/LPC ratios was considered – for the cord samples, this was the mother's BMI. BMI did not correlate significantly with the serum PC/LPC ratio for any of the groups (Fig. 2(C)). The PC/LPC ratio at 16 weeks of gestation did show a trend to being lower with increasing BMI suggestive of increased inflammation with obesity, which is unsurprising.^42^ The covariate effect of BMI on PC/LPC overall was found to be null but is significantly dependent on the stage of gestation (Fig. 2(D) and (E)).

### The PC/LPC ratio is significantly affected by GDM at 16 weeks but not 28 weeks of gestation

**Fig. 3:**
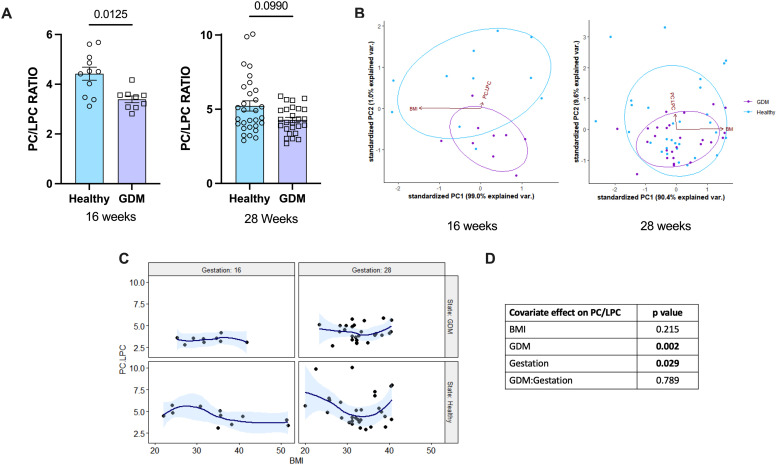
The PC/LPC ratio for healthy pregnant women *versus* pregnant women with GDM at 16 weeks and 28 weeks of gestation. Serum samples from healthy pregnant women (blue; *n* = 11 16W, *n* = 30 28W) and pregnant women with GDM (purple; *n* = 29 16W, *n* = 26 28W) were analysed on the MALDI-ToF as described in the materials and methods. (A) Statistical analysis was with a Mann–Whitney *t* test where *p* < 0.05 was significant. Times of gestation measured were: 16 weeks (*p* = 0.0125) and 28 weeks (*p* = 0.0990). To determine the covariate effect of the PC/LPC ratio, a PCA analysis was performed with PC/LPC and BMI (B), a regression analysis comparing gestational age, healthy state, and BMI on the effect of PC/LPC (C), and the 2-way ANCOVA analysis (D) which showed that GDM and gestational age had significant effect on PC/LPC, but not BMI.

To determine if this novel method for determining the PC/LPC ratio has potential as a diagnostic tool, sera from pregnancies complicated with GDM at 16 weeks and 28 weeks of gestation were analysed. Results were compared to those from healthy pregnancies at the same gestational timepoints (Table 2). The PC/LPC ratio in pregnancies complicated by GDM was decreased significantly at 16 weeks of gestation, but while also decreased at 28 weeks this was not significant (Fig. 3(A) and (B)). Studying the effect of covariation using a two-way ANCOVA, it showed that GDM does significantly influence the PC/LPC value (Fig. 3(C) and (D)).

**Table 2:** Descriptive statistics for the PC/LPC ratio for healthy pregnant women *versus* pregnant women with GDM at 16 weeks and 28 weeks of gestation

		Range	Mean ± SD
16 weeks	Healthy	3.1–5.7	4.4 ± 0.9
	GDM	2.8–4.2	3.4 ± 0.4

28 weeks	Healthy	2.9–10.1	5.2 ± 1.9
	GDM	2.7–5.9	4.2 ± 0.9

### The PC/LPC ratio is detectable in capillary samples, and can map out the menstrual cycle

**Fig. 4:**
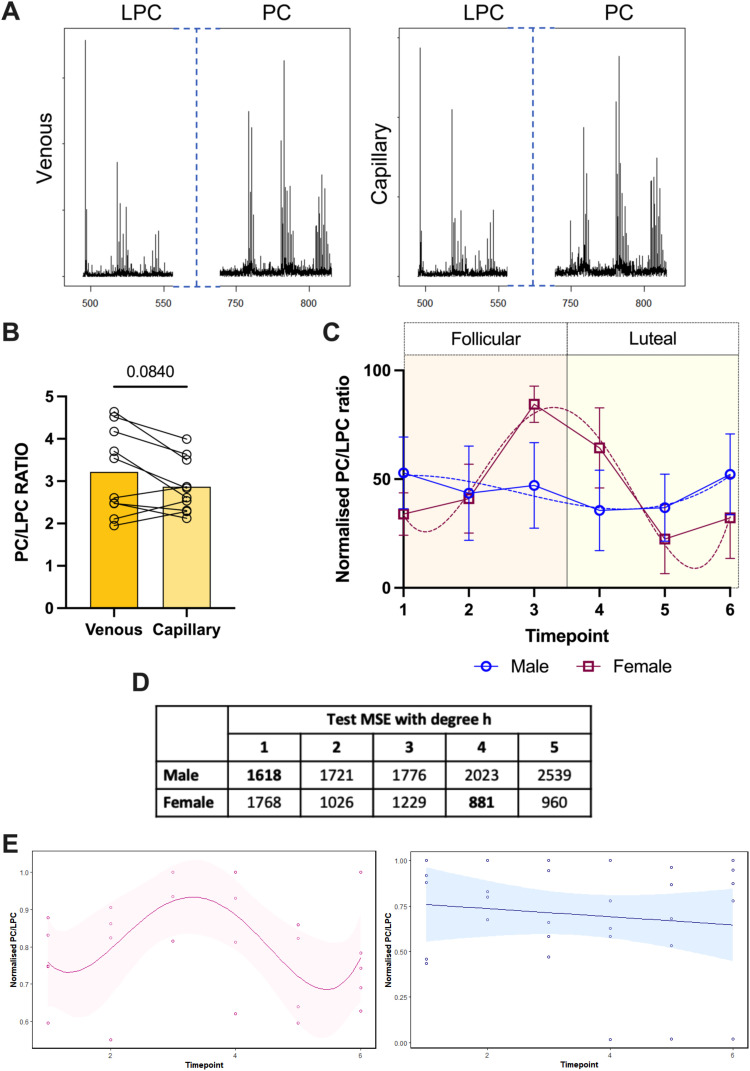
Detection of the PC/LPC ratio in capillary-derived sera and its fluctuation through the menstrual cycle. Capillary and venous blood was taken from the same healthy volunteers within the space of 30 min of collection (*n* = 10) and sera was analysed (*p* = 0.0840). Statistical analysis was with a Wilcoxon paired *t* test where *p* < 0.05 was significant. (A) Example spectra for venous *vs.* capillary sera. (B) The PC/LPC ratio was calculated for the sera (*p* = 0.0840). (C) Capillary-sera samples from healthy cycling non-pregnant women on no hormonal contraception (red; *n* = 5), and age-matched men (blue; *n* = 5) were analysed on the MALDI-ToF as described in the materials and methods. The PC/LPC ratio was normalised for each individual donor and plotted over 6 timepoints. (D) The PC/LPC ratio over time was expose to a model which tested the degree of polynomial. The lowest value corresponds to the most likely model to be fitted. (E) The plots for female (pink) and male (blue) with their preferred regression model (4th and 1st respectively).

Given that we had shown some translational potential in the measurement of PC/LPC ratio in pregnancy we then took next steps to evaluating the clinical utility of this approach. The first thing was to consider the use of capillary blood collected by finger-prick as more in keeping with a point of care approach. The use of finger-pricks for blood collection comes with many advantages. This includes, but is not limited to: ability to be conducted remotely,^43^ less invasive than venous blood sampling, and does not require a trained professional. Testing paired sera samples from healthy volunteers, the same species of PC and LPC were detectable in the capillary sera, as in the venous sera (Fig. 4(A)). There was no significant difference of the PC/LPC ratio between these sample collection types (Fig. 4(B)). We do acknowledge that there is some variability between capillary and venous blood, but we do not believe this will affect differences observed between groups.

We then sought a testing landscape to establish the utility of a finger-prick based approach to tracking PC/LPC ratio. We opted for the menstrual cycle as complementary to our initial work in pregnancy but offering the opportunity for a prospective analysis approach over a much shorter timescale. For this analysis, values for the PC/LPC ratio were normalised per donor as described in the materials and methods to account for inter-donor variability. Capillary blood was obtained, and serum prepared at 6 different time points over a 4 week period. The PC/LPC ratio remains steady over this timeframe for the male donors (linear regression) but shows cyclical variation for the females (polynomial 4th degree; Fig. 4(B)–(D)). All females reported a regular menstrual cycle and showed a PC/LPC ratio peak, representing lower inflammation, towards the end of the follicular phase and a drop in the PC/LPC ratio, indicating a pro-inflammatory response, during the luteal phase.

## Discussion

A lipidomic approach using intact serum to reveal PC/LPC was explored as a steppingstone to translating this approach to clinical utility. The focus was women's health including analysis related to pregnancy and the menstrual cycle – both times of dynamic change in the pro- and anti-inflammatory balance with dysregulation of this linked to adverse effects on pregnancy and fertility. Taking a method first developed and well validated using horse serum,^37^ we have shown that it is suitable for the analysis of human serum and can be used to reveal changes in the relative abundance of PC and LPC and provide insight into the inflammatory status of humans.

We first considered the utility of this mass spectrometry approach to track changes to the inflammatory balance in pregnancy. The bookends of pregnancy are marked with a pro-inflammatory load for implantation and labour; between these an escalating anti-inflammatory phenotype dominates the maternal systemic immune system.^29^ This is evidenced here by the minimal conversion of PC to LPC as gestation progresses suggesting an increasingly anti-inflammatory profile as pregnancy continues. Less conversion of PC to LPC would also help explain the protective effect of pregnancy against MS relapse, of which LPCs are considered to contribute. To complement this, a study has shown that LPC levels in pregnancy increase in the first term post conception but decrease to below not-pregnant levels in the second trimester, decreasing further in the third.^44^ In addition, other studies have shown increasing concentrations of PC in maternal plasma as gestation progresses,^45–47^ most likely to ensure an adequate supply for the fetus. This could also be due to the recommended supplementation of phosphatidylcholine during pregnancy, as studies have shown supplementation positively influences fetal development and child behaviour.^48^ High levels of PC are also required postnatally, as it is found rich in breast milk.^49^ All pregnant participants were scheduled for elective caesarean section for reasons such as breach presentation and previous emergency caesarean section and did not show any evidence of labour, so should not yet have tipped into the pro-inflammatory milieu associated with this. Future work should consider planned and emergency caesarean sections *versus* natural or induced labour to investigate what effect labour has on PC/LPC seeking to confirm the switch to a pro-inflammatory environment. The greater variability in PC/LPC metabolism towards the end of pregnancy could be linked to proximity to natural initiation of labour. Prospective analysis of women would address this and align to existing work that has suggested a metabolic clock of pregnancy that predicts gestational age and labour onset.^50^

Notably, fetal PC/LPC as measured in umbilical cord blood collected at delivery of the baby was comparable to non-pregnant rather than pregnant women. Increase in the production of PC from choline during pregnancy is by both the phosphatidylethanolamine *N*-methyltransferase (PEMT) and cytidine diphosphate (CDP)-choline pathways, but it is the PC produced by the PEMT pathway that is transferred selectively to the fetus in the third trimester.^51^ Dietary supplements of choline are recommended for pregnant women, due to this heightened requirement.^51^ High choline intake during pregnancy is vital for the woman's own needs, but also for placental function, neurodevelopment and epigenetic programming.^52^ In rat models, gestational supplementation of choline is linked with mitigation of alterations in genes associated with autism and schizophrenia.^53^ Early studies with humans have also realised that neuronal activity which is deficit with schizophrenia is improved with choline supplementation in pregnancy.^54^

Obesity is an inflammatory state, and studies have shown decreased LPC in obese subjects^55^ so the effects of donor BMI was considered here. There was no significant correlation between BMI and PC/LPC ratio, however, at 16 weeks of pregnancy, the negative correlation with BMI indicating increased LPC therefore increased inflammation was almost significant (*p* = 0.0566). This suggests that the effects of obesity on maternal inflammation might be greater in earlier gestation where it could be linked to increased risk of miscarriage^56^ and that any effects of maternal BMI on fetal development or maternal health might have their origins in the first half of pregnancy. As pregnancy progresses and maternal metabolism undergoes well-recognised characteristic changes,^22,27^ this might over-ride any overt effects of obesity. Clearly any further work warrants specific analysis of the intersection of maternal obesity and gestational age taking a prospective approach especially during the first trimester.

As our goal was to start evaluating the utility of this simple PC/LPC measurement as a potential biomarker we then considered if differences could be detected in women diagnosed with GDM. We chose this obstetric condition as elevated PC and reduced LPC has been described already in GDM,^32–34,57^ and our intact serum analysis approach using MALDI-ToF could perhaps provide a quick and cost-effective diagnosis of GDM. Samples were collected from women undergoing a glucose tolerance test (GTT) as part of the risk factor-based approach to screening for GDM in the UK. Women had fasted prior to GTT and providing a sample for our study and were undergoing testing at around either 16- or 28 weeks of gestation. PC/LPC was lower in women diagnosed with GDM at 16 weeks of pregnancy compared to their GTT-tested, GDM-negative counterparts. At 28 weeks of gestation there was no difference between the groups. Women are only typically tested at 16 weeks of gestation when they are at even higher risk of developing GDM, *i.e.*, GDM in a previous pregnancy or glucosuria, so this might account for the differences seen. Alternatively, as the effect of BMI was seen at 16 weeks, it again might that pregnancy *per se* eventually masks any difference in PC/LPC linked to donor health status and that this measure would only prove suitable as a biomarker of GDM if used earlier in pregnancy. Only a large prospective analysis using serum collected prior to 16 weeks of gestation and related to GDM outcomes alongside confounding variables such as obesity could test this.

Finally, on the path to considering the use of PC/LPC in a point of care setting, the utility of finger-prick blood collection as a viable option for measuring PC/LPC was considered. Given the practical considerations described above and that elevated PC in plasma and peritoneal fluid is associated strongly with endometriosis,^58^ we studied the menstrual cycle as a first step. There were clear differences in PC/LPC of men and women that were cyclically determined in women. Healthy men showed little variation in their PC/LPC when sampled 6 times over 4 weeks whereas healthy women who self-reported a normal menstrual cycle and no use of hormonal contraception showed fluctuations – peaking during the follicular phase, and the pattern follows that of estrogen (ESI,† Fig. S1). The cyclical PC/LPC pattern shown here might differ with menstrual disorders and offer a new biomarker for diagnosis. We have already initiated work to consider the effects of hormonal contraception and menstrual cycle disorders on natural fluctuation of PC/LPC in women of reproductive age in diseases related to the menstrual cycle, such as endometriosis. Current diagnostic measures for endometriosis are highly invasive, and the average time for diagnosis from onset of symptoms is a staggering 7.5 years.^59^ The method described in our study would allow for a framework of a less expensive and time-consuming model for diagnosis, particularly if a distinct timepoint after day 1 of cycle onset exhibits a more drastic deviation.

## Conclusion

Here we have highlighted a novel technique to quantify PC/LPC as a broad measure of inflammation to reveal the pro- *versus* anti-inflammatory balance quickly and effectively. The approach described has been used to show that the maternal systemic environment has an increasing anti-inflammatory status as gestation progresses, evidenced by increasing PC/LPC. The menstrual cycle study not only provides new insights into the inflammatory balance in the follicular and luteal phases but provides insight that the very earliest stages of pregnancy – aligned to elevated progesterone – are likely pro-inflammatory. Further work should use this study as a platform to further develop and validate the possible usage of PC/LPC as a diagnostic tool.

## Abbreviations

PC: Phosphatidylcholine
LPC: Lysophosphatidylcholine
GDM: Gestational diabetes mellitus
BMI: Body mass index
MALDI-ToF: Matrix-assisted laser desorption/ionisation – time of flight.

## Data availability

All data is contained within this manuscript.

## Conflicts of interest

There are no conflicts to declare.

## Supplementary Material

MO-019-D2MO00294A-s001

MO-019-D2MO00294A-s002
