# Supplementary material for: The dynamic inflammatory profile of pregnancy can be monitored using a novel lipid-based mass spectrometry technique
Source: Mol Omics. 2023 Mar 8;19(4):340–50. doi: 10.1039/d2mo00294a (PMC10167726; doi:10.1039/d2mo00294a)
Supplement: MO-019-D2MO00294A-s001 [file MO-019-D2MO00294A-s001.pdf]

Electronic Supplementary Material (ESI) for Molecular Omics.
This journal is © The Royal Society of Chemistry 2023

Supplemental figure 1: A graphical representation of the PC/LPC measurements in this study, in comparison to known progesterone and estrogen flux. Made using BioRender ©
