# Supplementary figures and images for: The dynamic inflammatory profile of pregnancy can be monitored using a novel lipid-based mass spectrometry technique
Source: Mol Omics. 2023 Mar 8;19(4):340–50. doi: 10.1039/d2mo00294a (PMC10167726; doi:10.1039/d2mo00294a)

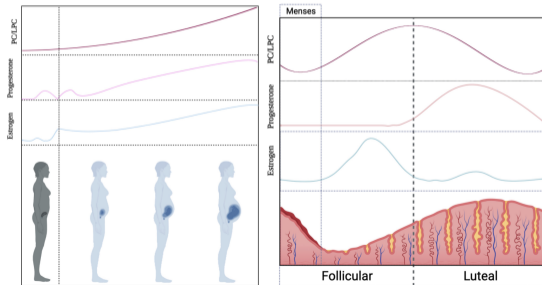

Supplement: MO-019-D2MO00294A-s002 [file MO-019-D2MO00294A-s002.pdf]
